# Prevalence and surgical management of pubic hypertrophy in hypospadias patients: results from a high-volume surgeon

**DOI:** 10.1590/S1677-5538.IBJU.2019.0267

**Published:** 2019-12-17

**Authors:** Marco Bandini, Sasha Sekulovic, Nikola Stanojevic, Bogdan Spiridonescu, Vladislav Pesic, Salvatore Sansalone, Milan Slavkovic, Alberto Briganti, Andrea Salonia, Francesco Montorsi, Rados Djinovic

**Affiliations:** 1 Sava Perovic Foundation, Center for Genito-Urinary Reconstructive Surgery, BelMedic General Hospital, Belgrade, Serbia;; 2 Division of Oncology and Unit of Urology, URI, IRCCS Ospedale San Raffaele, Vita-Salute San Raffaele University, Milan, Italy;; 3 Clinical Institute Fundeni, Center for Uronephrology and Renal Transplantation, Bucharest, Romania;; 4 Department of Experimental Medicine and Surgery, University of Tor Vergata, Rome, Italy

**Keywords:** Lipectomy, Pediatrics, Hypospadias, Hypertrophy

## Abstract

**Introduction:**

Pubic hypertrophy, defined as an abnormal and abundant round mass of fatty tissue located over the pubic symphysis, is frequently underestimated in patients with hypospadias. We examined the prevalence of this condition, as well as the outcomes associated with its surgical treatment.

**Material and methods:**

Within 266 hypospadias patients treated at our clinic, we assessed the prevalence of pubic hypertrophy, and we schematically described the surgical steps of pubic lipectomy. Multivariable logistic regression (MLR) tested for predictors of pubic hypertrophy. Finally, separate MLRs tested for predictors of fistula and any complications after pubic lipectomy.

**Results:**

Of 266 hypospadias patients, 100 (37.6%) presented pubic hypertrophy and underwent pubic lipectomy. Patients with pubic hypertrophy more frequently had proximal hypospadias (44 vs. 7.8%), disorders of sex development (DSD) (10 vs. 0.6%), cryptorchidism (12 vs. 2.4%), and moderate (30°-60°) or severe (>60°) penile curvature (33 vs. 4.2%). In MLR, the location of urethral meatus (proximal, Odds ratio [OR]: 10.1, p<0.001) was the only significant predictor of pubic hypertrophy. Finally, pubic lipectomy was not associated with increased risk of fistula (OR: 1.12, p=0.7) or any complications (OR: 1.37, 95% CI: 0.64-2.88, p=0.4) after multivariable adjustment.

**Conclusions:**

One out of three hypospadias patients, referred to our center, presented pubic hypertrophy and received pubic lipectomy. This rate was higher in patients with proximal hypospadias suggesting a correlation between pubic hypertrophy and severity of hypospadias. Noteworthy, pubic lipectomy was not associated with increased risk of fistula or any complications.

## INTRODUCTION

Hypospadias is a congenital defect of the male genitalia characterized by an ectopic location of urethral meatus on the ventral side of the penis, proximal to the tip of the glans penis. It is generally accepted that genetic, hormonal and environmental factors play a major role in the etiology of hypospadias (1). All these factors seem also to play a role in the etiology of the other penile abnormalities, which are frequently associated with hypospadias. Such included penile curvature (2), undescended testis (3), underdeveloped foreskin (4) and penoscrotal transposition (5). All these conditions have been well described in the literature. In contrast, there is a paucity of information with regards to pubic hypertrophy (6) in hypospadias patients.

**Figure 1:**
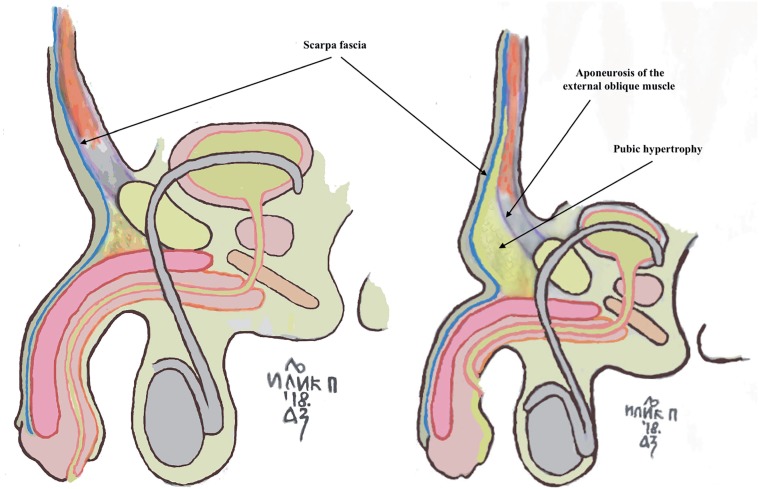
Comparison between physiological and pathological suprapubic fat tissue in patients with hypospadias.

**Supplementary Figure 1:**
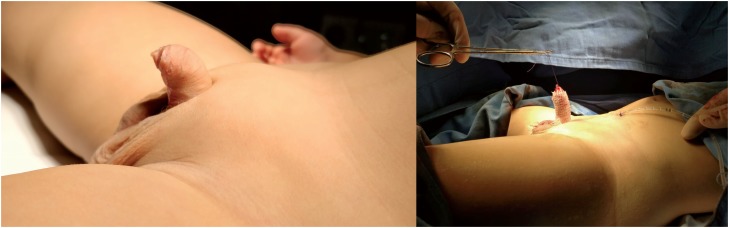
Before and after pubic lipectomy in patient with pubic hypertrophy. The abundant suprapubic fat mimics the mons pubic and it is hiding the penis for half of his length.

Pubic hypertrophy is characterized by an abundant round mass of fatty tissue located over the pubic symphysis (Figure 1 and supplementary Figure 1). More specifically, it appears as hypertrophic adipose tissue located between the fascia of Scarpa and the aponeurosis of the external oblique muscle, that simulates the mons pubis. Additionally, pubic hypertrophy is typically associated with a lack of firm attachments between the Buck and the Dartos fascia with the pubic bones, causing in that way, the instability of the penis (7). To date, its presence has been underreported in hypospadias patients, in whom the attention has been more frequently focused on the urethral and penile malformations. Nevertheless, the excess of fatty tissue in this area may lead to further aesthetic and functional problems during child development, which may ultimately end in a condition noted as buried penis (8).

To the best of our knowledge, no study has previously described the incidence and the treatment of pubic hypertrophy in hypospadias patients. With this in mind, we aimed to report the prevalence of this aesthetic malformation in our case series, which included only hypospadias patients. Furthermore, we described the surgical technique adopted for the correction of the pubic hypertrophy. Finally, we reported the postoperative outcomes and the complications associated with its surgical treatment.

## PATIENTS AND METHODS

### Study population

The analysis population relied on the hypospadias database of the BelMedic clinic in Belgrade. This contemporary database includes data retrospectively gathered between January 2013 and April 2018. All included patients were referred to our clinic for hypospadias repair and all were operated by the same surgeon (RD). Criteria for patient selection included: primary or failed hypospadias repair. Overall, hypospadias repairs were carried using different urethroplasty techniques such as tubularized incised plate urethroplasty (TIP) (9), Mathieu urethroplasty (10), direct tubularization of the urethra, two-stage urethroplasty with buccal mucosa graft or labial mucosa graft (11) and others (12). Seven patients did not receive urethroplasty since they presented hypospadias-sine-hypospadias (13). Those patients underwent to glansplasty and/or dorsal plication in order to correct the congenital defect and to achieve the penile straightening. Of all 266 patients referred to our clinic for hypospadias repair, 100 presented pubic hypertrophy and received pubic lipectomy.

### Pubic lipectomy-surgical technique

**Figure 2:**
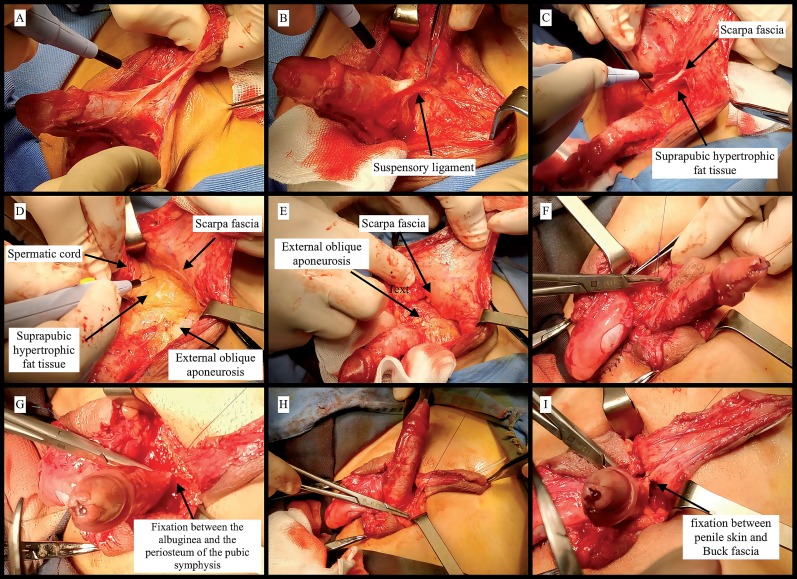
This image presents the step-by-step procedure for the pubic lipectomy. (A): Penile degloving. (B): Dissection of the fundiform and suspensory ligaments. (C): Detachment of the suprapubic fat from the Scarpa fascia. (D): The hypertrophic fat tissue is dissected, meanwhile the two spermatic cords are laterally traced. (E): The external oblique aponeurosis is visible at the end of the resection. (F-G-H): Fixation of the Buck fascia to the periosteum of the pubic symphysis with absorbable 0-4 polydioxanone suture at 12 o'clock and fixation of the penis to the aponeurosis of the external oblique muscle at 3 and 9 o'clock. (I): Fixation between the penile skin at the base of the penis and the Buck fascia at 12, 3, 6 and 9 o'clock.

We placed a stay suture through the glans for traction. A skin incision was done around the hypospadic meatus and continued towards the coronal level. Penile degloving was performed carefully in order to prevent injury of divergent spongiosal tissue and possible excessive bleeding. The suspensory and fundiform ligaments were divided freeing of the skin and the fascia of Scarpa from the body of the penis; such was made in order to get access to the fatty tissue to be excised. Care was taken to avoid damage to the nerves and vessels of the penis which enter the corpora of the penis at the 2 and 10 o'clock positions. The excision of the fatty tissue was done below the Scarpa fascia in order to avoid any damage of the blood vessels and the nerves directed to the penile skin. This step was facilitated by using the front and back lighting technique (4), which allowed the identification of the skin vessels that run within the Scarpa fascia. Subsequently, the suprapubic fat was detached from the aponeurosis of the external oblique muscle, which represented the posterior limit of the dissection. Here, the hypertrophic fat was traced by surgical forceps and the dissection was performed using the electrocautery, which allowed the coagulation of small communicant vessels. It should be noted that the spermatic cords, which run into the adipose tissue, were carefully isolated and laterally traced to avoid any injury during the resection of the suprapubic fat. The procedure was completed with the fixation of the albuginea to the periosteum at the symphysis pubis with two absorbable 4-0 polydioxanone suture at 12 o'clock. Two more sutures fixed the penis to the aponeurosis of the external oblique muscle at 3 and 9 o'clock. The penile skin at the base of the penis was than fixed to the Buck fascia by 4 sutures at 12, 3, 6 and 9 o'clock (Figure-2). Urethroplasty was subsequently performed using different surgical techniques depending on the location of the urethral meatus and on the anatomical characteristics. In case of penile curvature, the straightening of the penis was also achieved through relaxing incisions or plications of the tunica albuginea depending on the severity of the curvature (14). Finally, we fixed the testis to the scrotal skin bilaterally and we placed a corrugated drain through the scrotum to facilitate the drainage of the suprapubic space. Testis fixation and drain were removed after 5 days from the operation. We applied Coban 3M, USA dressing around the penis in a stretched position and suprapubic gauzes for compression. Suprapubic catheter and urethral stent were left in place for 14 days.

### Surgical outcomes after pubic lipectomy

The primary evaluated surgical outcome was the incidence of fistula after pubic lipectomy. Fistula was defined as an abnormal opening of the perineal or penile urethra that occurred after the hypospadias repair. The secondary evaluated surgical outcome was the incidence of any complications after pubic lipectomy. For the definition of any complications we included the presence of fistula, diverticulum, hematoma, residual curvature, urethral stricture, residual hypospadias, skin necrosis, lymphoedema, meatal stenosis and meatal dehiscence.

### Statistical analyses

Descriptive statistics included frequencies and proportions for categorical variables. Medians, and interquartile ranges (IQR) were reported for continuously coded variables. For data representation purpose, patients were stratified in two groups: 1) who had pubic hypertrophy and received pubic lipectomy and 2) who did not have pubic hypertrophy and consequently did not receive pubic lipectomy. The statistical significance of differences in medians and proportions between the two groups was respectively tested with the Kruskal-Wallis and Chi-square tests. Subsequently, univariable and multivariable logistic regression (MLR) models (15) tested for predictors of pubic hypertrophy. Finally, we relied on two sets of MLR models to test the relationship between pubic lipectomy and fistula, as well as between pubic lipectomy and any complication. All statistical tests were two-sided with a level of significance set at p <0.05. Analyses were performed using the R software environment for statistical computing and graphics.

## RESULTS

### Study population

Between January 2013 and April 2018, we retrospectively identified 266 hypospadias patients that were suitable for the study purposes (Table-1). All patients were Caucasian and the median age at surgery was 24 months (IQR: 15.6-42.9). Hundred individuals (37.6%) presented pubic hypertrophy and received pubic lipectomy. Patients with pubic hypertrophy had more frequently proximal hypospadias (44 vs. 7.8%, p <0.001), disorders of sex development (DSD) (10 vs. 0.6%, p <0.001), cryptorchidism (12 vs. 2.4%, p=0.003) and moderate (30°-60°) or severe (>60°) grade of curvature (33 vs. 4.2%, p <0.001). Overall, single-stage hypospadias repair was preferred (79.7 vs. 20.3%) over the two-stage repair. Conversely, patients with pubic hypertrophy more frequently received two-stage repair (45 vs. 5.4%, p <0.001).

**Table 1:** General patient characteristics of 266 individuals referred to our clinic for hypospadias, between January 2013 and April 2018.

Variables		Overall	No pubic lipectomy (n= 166)	Pubic lipectomy (n= 100)	p values
Age at surgery (months)	Median	24	24	24	0.4
	Range	15.6-42.9	16.8-45.3	15.6-38.7	
Number of surgical stages	1	212 (79.7)	157 (94.6)	55 (55)	<.001
	2	54 (20.3)	9 (5.4)	45 (45)	
Location of the meatus	Distal	146 (54.9)	117 (70.5)	29 (29)	<.001
	Midshaft	63 (23.7)	36 (21.7)	27 (27)	
	Proximal (peno-scrotal, scrotal, perineal)	57 (21.4)	13 (7.8)	44 (44)	
Previously operated for hypospadias	0	211 (79.3)	137 (82.5)	74 (74)	0.08
	1	53 (19.9)	27 (16.3)	26 (26)	
Penile curvature at presentation	<30°	45 (16.9)	38 (22.9)	7 (7)	<.001
	30°-60°	165 (62)	112 (67.5)	53 (53)	
	>60°	56 (21.1)	16 (9.6)	40 (40)	
DSD	No	255 (95.9)	165 (99.4)	90 (90)	<.001
	Yes	11 (4.1)	1 (0.6)	10 (10)	
Urethroplasty technique	TIP	119 (44.7)	84 (50.6)	35 (35)	<.001
	Mathieu	43 (16.2)	35 (21.1)	8 (8)	
	hypospadias-sine-hypospadias	7 (2.6)	5 (3)	2 (2)	
	Other	7 (2.6)	6 (3.6)	1 (1)	
	Tubularized	25 (9.4)	17 (10.2)	8 (8)	
	Use of buccal mucosa graft	58 (21.8)	13 (7.8)	45 (45)	
	Use of labial mucosa graft	7 (2.6)	6 (3.6)	1 (1)	
Curvature management at 1° step	None	14 (5.3)	14 (8.4)	0 (0)	<.001
	Cordee release and/or ventro-tunical attenuation	61 (22.9)	18 (10.8)	43 (43)	
	Plication	191 (71.8)	134 (80.7)	57 (57)	
Curvature management at 2° step	None	222 (83.5)	158 (95.2)	64 (64)	<.001
	Cordee release and/or ventro-tunical attenuation	1 (0.4)	0 (0)	1 (1)	
	Plication	43 (16.2)	8 (4.8)	35 (35)	
Skin reconstruction	Direct ventral closure	216 (81.2)	142 (85.5)	74 (74)	0.03
	Use of flaps or grafts	50 (18.8)	24 (14.5)	26 (26)	
Position of the testicles	Scrotal	250 (94)	162 (97.6)	88 (88)	0.003
	Cryptorchidism	16 (6)	4 (2.4)	12 (12)	
Complications		Overall	No pubic lipectomy (n= 166)	Pubic lipectomy (n= 100)	p values
Fistula	No	225 (84.6)	146 (88.6)	79 (79)	0.06
	Yes	40 (15)	19 (11.4)	21 (21)	
Any complications	No	213 (80.1)	141 (84.9)	72 (72)	0.02
	Yes	53 (19.9)	25 (15.1)	28 (28)	

### Univariable and multivariable logistic regression models testing for predictors of pubic hypertrophy

In univariable logistic regression model, location of the meatus (midshaft Odds ratio [OR]: 3.03, 95% confidence interval [CI]: 1.59-5.79, p <0.001; proximal OR: 13.66, 95% CI: 6.68-29.6, p <0.001), location of the testicles (cryptorchidism OR: 5.52, 95% CI: 1.86-20.22, p=0.004) and severity of the curvature (30°-60° OR: 2.57, 95% CI: 1.13-6.62, p=0.03, >60° OR: 13.57, 95% CI: 5.28-39.19, p <0.001) were all associated with higher rates of pubic hypertrophy, while age at surgery was not (OR: 1.00, 95% CI: 0.991.00, p=0.4). After multivariable adjustment, only the location of the meatus (midshaft OR: 3.28, 95% CI: 1.66-6.55, p <0.001; proximal OR: 10.09, 95% CI: 3.51-31.91, p <0.001) remained statistically significantly associated with higher rates of pubic hypertrophy, while curvature of 30°-60° (OR: 2.53, 95% CI: 1.08-6.74, p=0.045) was only marginally associated (Table-2).

**Table 2:** Univariable and Multivariable logistic regression models testing for predictors of pubic hypertrophy within 266 patients treated for hypospadias.

	Univariable				Multivariable			
Variables	OR	95% CI	95% CI	P values	OR	95% CI	95% CI	P values
Age at surgery	1.00	0.99	1.00	0.4				
Location of the meatus (Ref. distal)	Ref.	Ref.	Ref.	Ref.	Ref.	Ref.	Ref.	Ref.
Midshaft	3.03	1.59	5.79	<.001	3.28	1.66	6.55	<.001
Proximal	13.66	6.68	29.60	<.001	10.09	3.51	31.91	<.001
Position of the testicles (Ref. scrotal)	Ref.	Ref.	Ref.	Ref.	Ref.	Ref.	Ref.	Ref.
Cryptorchidism	5.52	1.86	20.22	0.004	3.30	0.93	13.94	0.07
Curvature (Ref. <30°)	Ref.	Ref.	Ref.	Ref.	Ref.	Ref.	Ref.	Ref.
Curvature 30°-60°	2.57	1.13	6.62	0.03	2.53	1.08	6.74	0.045
Curvature >60°	13.57	5.28	39.19	<.001	2.97	0.82	10.97	0.09

### Univariable and multivariable logistic regression models testing for fistula and any complications in patients treated with or without pubic lipectomy

Median follow-up was 34 months (IQR: 17-45months). Overall, patients treated with pubic lipectomy presented higher rates of fistula (21.0 vs. 11.4%) and any complications (28 vs. 15.1%) compared to patients who did not receive pubic lipectomy. Similarly, in univariable analyses pubic lipectomy was associated with higher risk of fistula (OR: 2.04, 95% CI: 1.04-4.05, p=0.04) and higher risk of any complications (OR: 2.19, 95% CI: 1.19-4.06, p=0.01) (Table-3). Conversely, after multivariable adjustment, pubic lipectomy was no longer associated with increased risk of fistula (OR: 1.12, 95% CI: 0.46-2.64, p=0.7) and any complications (OR: 1.37, 95% CI: 0.64-2.88, p=0.4) (Table-4).

**Table 3:** Univariable and multivariable logistic regression models testing for fistula in patients treated with or without pubic lipectomy, within 266 patients treated for hypospadias.

	Univariable				Multivariable			
Variables	OR	95% CI	95% CI	P values	OR	95% CI	95% CI	P values
Age at surgery	1.00	0.99	1.01	0.7				
Location of the meatus (Ref. distal)	Ref.	Ref.	Ref.	Ref.	Ref.	Ref.	Ref.	Ref.
Midshaft	2.50	1.09	5.73	0.03	1.94	0.77	4.73	0.1
Proximal	2.79	1.21	6.41	0.02	0.14	0.01	0.93	0.06
Position of the testicles (Ref. scrotal)	Ref.	Ref.	Ref.	Ref.				
Cryptorchidism	1.32	0.29	4.35	0.6				
ASSOCIATE DSD (Ref. no DSD)	Ref.	Ref.	Ref.	Ref.				
ASSOCIATE DSD	1.26	0.19	5.14	0.7				
Curvature (Ref. <30°)	Ref.	Ref.	Ref.	Ref.	Ref.	Ref.	Ref.	Ref.
Curvature 30°-60°	0.99	0.37	3.13	0.9	0.92	0.33	2.98	0.8
Curvature >60°	3.49	1.24	11.45	0.02	2.78	0.49	15.47	0.2
Lipectomy (Ref. no lipectomy)	Ref.	Ref.	Ref.	Ref.	Ref.	Ref.	Ref.	Ref.
Lipectomy	2.04	1.04	4.05	0.04	1.12	0.46	2.64	0.7
Previous hypospadias surgery (Ref. no previous surgery)	1.40	0.61	2.99	0.4				
Number of stages (Ref. 1 stage)	Ref.	Ref.	Ref.	Ref.				
Two stages	3.76	1.81	7.71	0.0003	9.32	1.44	78.66	0.02
Use of flaps or grafts (Ref. no use of grafts or flaps)	Ref.	Ref.	Ref.	Ref.				
Use of flaps or grafts	1.30	0.55	2.85	0.5				

**Table 4:** Univariable and multivariable logistic regression models testing for any complications in patients treated with or without pubic lipectomy, within 266 patients treated for hypospadias.

		Univariable				Multivariable		
Variables	OR	95% CI	95% CI	P values	OR	95% CI	95% CI	P values
Age at surgery	1.00	0.99	1.00	0.6				
Location of the meatus (Ref. distal)	Ref.	Ref.	Ref.	Ref.	Ref.	Ref.	Ref.	Ref.
Midshaft	2.47	1.18	5.17	0.02	2.02	0.92	4.39	0.07
Proximal	2.84	1.34	6.00	0.006	0.40	0.08	0.72	0.02
Position of the testicles (Ref. scrotal)	Ref.	Ref.	Ref.	Ref.				
Cryptorchidism	1.91	0.58	5.53	0.24				
Curvature (Ref. <30°)	Ref.	Ref.	Ref.	Ref.	Ref.	Ref.	Ref.	Ref.
Curvature 30°-60°	1.02	0.43	2.70	0.9	0.95	0.39	2.56	0.9
Curvature >60°	3.02	1.18	8.48	0.03	1.93	0.40	9.15	0.4
Lipectomy (Ref. no lipectomy)	Ref.	Ref.	Ref.	Ref.	Ref.	Ref.	Ref.	Ref.
Lipectomy	2.19	1.19	4.06	0.01	1.37	0.64	2.88	0.4
ASSOCIATE DSD (Ref. no DSD)	Ref.	Ref.	Ref.	Ref.				
ASSOCIATE DSD	1.54	0.33	5.53	0.5				
Previous hypospadias surgeries (Ref. no previous surgery)	Ref.	Ref.	Ref.	Ref.				
Previous hypospadias surgeries	1.58	0.77	3.15	0.2				
Number of stages (Ref. 1 stage)	Ref.	Ref.	Ref.	Ref.	Ref.	Ref.	Ref.	Ref.
Two stages	3.19	1.63	6.20	0.0006	3.91	0.73	23.42	0.1
Use of flaps or grafts (Ref. no use of grafts or flaps)	Ref.	Ref.	Ref.	Ref.				
Use of flaps or grafts	1.17	0.53	2.41	0.6				

## DISCUSSION

The association between pubic hypertrophy and hypospadias has not been previously described in the literature, where these two malformations have been always considered two distinct issues and managed in separate moments. Indeed, in the plethora of studies dealing with treatment of abundant suprapubic tissue, few examined the prevalence of previous hypospadias repair and none of them analyzed the correlation between the two malformations. For example, in 2004, Frenkl et al. (16) described a surgical technique for treatment of abundant suprapubic tissue and buried penis in 79 individuals referred to their institution. Of all 79 patients, 6 (7.6%) reported a history of previous hypospadias repair. However, no correlation was investigated between the two conditions. Similarly, Hadidi et al. (8) reported a case series of 12 patients with abundant suprapubic tissue and buried patients operated at their institution. Here, 5 out of 12 (42%) patients had a history of previous hypospadias repair. However, also in this study no correlation was investigated between the two conditions in spite the large prevalence. Based on the lack of data correlating hypospadias and pubic hypertrophy, we aimed to report the prevalence of pubic hypertrophy in our hypospadias cohort of patients and the association between pubic hypertrophy and the severity of hypospadias (i.e. location of the urethral meatus) (17). Furthermore, we schematically described all the steps of pubic lipectomy and finally, we evaluated the rates of fistula, as well as the rate of any complications in patients underwent pubic lipectomy.

The first noteworthy result yielded by our study was that more than 30% of our hypospadias patients exhibited pubic hypertrophy. Moreover, we also found that the proximal location of the ectopic urethral meatus, as well as the severity of the curvature, the incidence of DSD and the cryptorchidism were all more frequently manifested in patients with pubic hypertrophy. On top of that, the severity of hypospadias (i.e. proximal hypospadias) was multivariably associated with pubic hypertrophy. These results supported our hypothesis that pubic hypertrophy and hypospadias were frequently associated and that the incidence of pubic hypertrophy might be related with the severity of the urethral malformation. On the other hand, lack of data granularity hampered the demonstration of this correlation, which remains only an author speculation derived from data analyses.

Secondly, our study is also noteworthy because we are the first who describe a surgical technique for treatment of pubic hypertrophy in hypospadias patients. In our study, all 100 patients with pubic hypertrophy underwent pubic lipectomy using the described technique. It is of interest to know that no severe complications were recorded after surgery. It is also important to know that the most challenging step was the dissection of the suprapubic fat tissue from the fascia of Scarpa. In this phase, the surgeon must avoid any possible damage of the Scarpa fascia which should be left intact. This is of extremely importance since the Scarpa fascia is crossed by vessels and nerves directed to the skin of the penis. In consequence, its preservation is necessary to prevent the necrosis of the penile skin. Another important step is the bilateral isolation of the spermatic cords. Indeed, they represent the only structures that passed through the suprapubic fat. In consequence, both the spermatic cords should be isolated and laterally traced before starting the resection of the hypertrophic tissue to avoid any damage. Furthermore, because we mobilized the spermatic cords bilaterally to allow the resection of the suprapubic fat, we preferred to fix the testis in order to avoid their retraction outside the scrotum during the healing process.

Thirdly, we found that patients treated with pubic lipectomy more frequently reported postoperative fistula and any complications compared to patients who did not receive pubic lipectomy. At the same time, patients who underwent pubic lipectomy more frequently received two-stage repair surgery because of proximal hypospadias. Furthermore, they more frequently had albuginea plications because of severe curvature. Finally, they more frequently required the use of flaps and/or grafts for skin reconstruction. Taken together, the univariable correlation between fistula and pubic lipectomy, as well as between any complications and pubic lipectomy might be related to the higher complexity of the hypospadias surgery that was carried in patients with several malformations. In other words, we found that the greater the number of malformations, the more complex the surgery, and the higher the frequency of complications. This hypothesis was further supported by the results of our MLR, where pubic lipectomy was no longer associated with higher rates of fistula or any complications. Conversely, the location of the ectopic urethral meatus and the number of surgical stages, which were indirectly associated with the severity of the disease, were the only statistically significant predictors in the two MLR models.

Our study is not devoid of limitations rather than its retrospective design. First, we acknowledge that the small sample size of our study may limit the generalizability of its findings. Second, we also acknowledge that in our referral center, we treat more frequently complex cases of hypospadias. In consequence, the rate of severe hypospadias, as well as the rate of pubic hypertrophy might be higher than in a non-referral center. The third main limitation is represented by the short follow-up of the study (median 34 months), which may have limited the number of event or complications recorded after surgery. This limitation might have been overcome by including more historical patients. However, we decided a priory to consider only contemporary patients to avoid the inclusion of patients possibly operated with surgical techniques no longer used, introducing a more important bias to our study. Fourth, we lack of an objective definition of pubic hypertrophy. Indeed, the indication for pubic lipectomy was based on the aesthetical appearance of the pubis and on the absence of firm attachments between the Buck fascia and the pubic bone. In other words, we relied on subjective criteria that were both susceptible of the personal judgment of the surgeon. However, no other criteria were considered suitable for the indication of pubic lipectomy and we based our decision only on these two factors. Indeed, pubic hypertrophy is an aesthetical malformation, and as many other aesthetical malformations, it is not numerically quantifiable. Last, our study was not able to account for other biochemical variables that might explain the correlation between pubic hypertrophy and hypospadias. This said, biochemical data are neither available nor will be available due to the lack of ongoing study evaluating the biochemical correlation between pubic hypertrophy and hypospadias. In consequence, despite no strong conclusions can be derived from our analyses, our study represents the largest and most contemporary source of evidences that support the correlation between hypospadias and pubic hypertrophy and between pubic hypertrophy and the severity of hypospadias.

## CONCLUSIONS

According to our retrospectively data, we found that one out of three hypospadias patients presented pubic hypertrophy at the time of surgery. This rate was remarkably higher in patients with proximal hypospadias suggesting a correlation between pubic hypertrophy and the severity of hypospadias. Pubic lipectomy should be considered in patients with pubic hypertrophy in order to remove the abundant suprapubic fat tissue. Noteworthy, pubic lipectomy did not show to increase the risk of fistula or any complications, when it was carried during hypospadias repair.

## ABBREVIATIONS

MLR: Multivariable logistic regression
DSD: Disorders of sex development
TIP: Tubularized incised plate urethroplasty
IQR: Interquartile ranges
OR: Odds Ratio
CI: Confidence Interval

## References

[1] 1. Shih EM, Graham JM Jr. Review of genetic and environmental factors leading to hypospadias. Eur J Med Genet. 2014;57:453-63. 10.1016/j.ejmg.2014.03.003 24657417

[2] 2. Perovic SV, Djordjevic ML, Djakovic NG. A new approach to the treatment of penile curvature. J Urol. 1998;160(3 Pt 2):1123-7. 10.1097/00005392-199809020-00042 9719290

[3] 3. Sekaran P, O'Toole S, Flett M, Cascio S. Increased occurrence of disorders of sex development, prematurity and intrauterine growth restriction in children with proximal hypospadias associated with undescended testes. J Urol. 2013;189:1892-6. 10.1016/j.juro.2012.11.047 23159278

[4] 4. Perovic SV, Radojicic ZI. Vascularization of the hypospadiac prepuce and its impact on hypospadias repair. J Urol. 2003;169:1098-100. 10.1097/01.ju.0000052820.35946.99 12576861

[5] 5. Perovic S, Vukadinovic V. Penoscrotal transposition with hypospadias: 1-stage repair. J Urol. 1992;148:1510-3. 10.1016/s0022-5347(17)36952-5 1433560

[6] 6. Bandini M, Sekulovic S, Stanojevic N, et al. Prevalence and surgical management of pubic hypertrophy in hypospadias patients: Results from a high-volume surgeon. Eur Urol Suppl. 2019;18:e754-e755. 10.1590/S1677-5538.IBJU.2019.0267 PMC6909876 31808413

[7] 7. Alter GJ. Pubic contouring after massive weight loss in men and women: correction of hidden penis, mons ptosis, and labia majora enlargement. Plast Reconstr Surg. 2012;130:936-47. 10.1097/PRS.0b013e318262f57d 23018703

[8] 8. Hadidi AT. Buried penis: classification surgical approach. J Pediatr Surg. 2014;49:374-9. 10.1016/j.jpedsurg.2013.09.066 24528990

[9] 9. Snodgrass W, Bush N. Tubularized incised plate proximal hypospadias repair: Continued evolution and extended applications. J Pediatr Urol. 2011;7:2-9. 10.1016/j.jpurol.2010.05.011 20598641

[10] 10. Hueber PA, Antczak C, Abdo A, Franc-Guimond J, Barrieras D, Houle AM. Long-term functional outcomes of distal hypospadias repair: a single center retrospective comparative study of TIPs, Mathieu and MAGPI. J Pediatr Urol. 2015;11:68. 10.1016/j.jpurol.2014.09.011 25824882

[11] 11. Bracka A. Hypospadias repair: the two-stage alternative. Br J Urol. 1995;76(Suppl 3):31-41. 10.1111/j.1464-410x.1995.tb07815.x 8535768

[12] 12. Djordjevic ML, Majstorovic M, Stanojevic D, Bizic M, Kojovic V, Vukadinovic V, et al. Combined buccal mucosa graft and dorsal penile skin flap for repair of severe hypospadias. Urology. 2008;71:821-5. 10.1016/j.urology.2007.12.004 18336884

[13] 13. Singh S, Rawat J, Kureel SN, Pandey A. Chordee without hypospadias: Operative classification and its management. Urol Ann. 2013;5:93-8. 10.4103/0974-7796.110005 PMC3685753 23798865

[14] 14. Bandini M, Sekulović S, Dangi AD, et al. Corporeal penile curvature (CPC) and surgical complications in hypospadias repairs: Associations and outcomes. Eur Urol Suppl. 2019;18:e752.

[15] 15. Van Smeden M, de Groot JA, Moons KG, Collins GS, Altman DG, Eijkemans MJ, et al. No rationale for 1 variable per 10 events criterion for binary logistic regression analysis. BMC Med Res Methodol. 2016;16:163. 10.1186/s12874-016-0267-3 PMC5122171 27881078

[16] 16. Frenkl TL, Agarwal S, Caldamone AA. Results of a simplified technique for buried penis repair. J Urol. 2004; 171 (2 Pt 1):826-8. 10.1097/01.ju.0000107824.72182.95 14713835

[17] 17. M. Bandini, S. Sekulovic, N. Stanojevic, M. Slavkovic, B. Spiridonescu, A.D. Dangi, et al. Hypospadias complexity score (HCS): A new tool for predicting operating time and complications in hypospadias surgery. Eur Urol Suppl. 2019, 18,1,e741.

